# Simultaneous Evaluation of the Influence of* Panax ginseng* on the Pharmacokinetics of Three Diester Alkaloids after Oral Administration of Aconiti Lateralis Radix in Rats Using UHPLC/QQQ-MS/MS

**DOI:** 10.1155/2018/6527549

**Published:** 2018-12-09

**Authors:** Liang Yang, Yuguang Wang, Guangyao Huang, Jian Li, Zhaoyan Zhang, Zengchun Ma, Yue Gao

**Affiliations:** Beijing Institution of Radiation Medicine, Beijing 100850, China

## Abstract

**Objectives:**

To investigate whether* Panax ginseng* (*P. ginseng*) could affect the metabolism of Diester Alkaloids (DAs) derived from Aconiti Lateralis Radix in vivo.

**Methods and Results:**

24 male Sprague-Dawley rats were randomized for 7-day treatment with* P. ginseng* (low, middle, and high), or vehicle. Aconiti Lateralis Radix was administered orally to each group on the 8th day. Plasma samples were collected, and Xevo TQ-S was used to detect the concentration of aconitine, mesaconitine, and hypaconitine in plasma. We describe a fast and reproducible method to detect the concentration of aconitine, mesaconitine, and hypaconitine in plasma. Compared to the control group, the AUC_(0-t)_ of three DAs increased in both the middle and high dosing groups. The Vz/F of three DAs in these groups as well as the CLz/F of aconitine in all* P. ginseng* groups and the CLz/F of mesaconitine and hypaconitine in* P. ginseng* middle and high groups were decreased compared to the control group.

**Conclusion:**

Orally administrated* P. ginseng* potentially inhibits the metabolism of DAs from Aconiti Lateralis Radix in rats.

## 1. Introduction

*Panax ginseng C. A. Mey* (referred as* P. ginseng* in this study) and Aconiti Lateralis Radix are two traditional Chinese medicines that have a long history of successful application in the clinic [1–3].

*P. ginseng* has been used for the treatment of various diseases such as liver dysfunction [4], hypertension [5], and cerebrovascular disease [6] in China, Japan, and Korea. Various ginsenosides such as Rg1, Rb1, Rc, Rd, Rg3, Rh1, and Rf have all been found to be important constituents in* P. ginseng* extracts [7–10]. Aconiti Lateralis Radix is traditional Chinese medicine famed for its toxicity and contains a range of diverse alkaloids such as aconitine, mesaconitine, hypaconitine, and benzoylaconitine among many others [11]. Aconitine, mesaconitine, and hypaconitine, which are classed as DAs, have been shown to be the major toxic components in Aconiti Lateralis Radix and have been known to exert both potent cardiac toxicity and neurotoxicity in several studies [12, 13].

Routes of administration vary when a combination of* P. ginseng* with Aconiti Lateralis Radix is required, including the two traditional routes, by injection and by mouth [1, 2]. These routes are commonly used for a range of disorders, most notable in the treatment of heart failure and rescuing hemorrhagic shock [14–19]. Although this treatment is often successful, overdose is common among patients. New measures to reduce this risk are now in place including more strictly controlled usage to limit cardiac toxicity and neurotoxicity. To date, the mode of action of this toxicity is unknown; however, several hypotheses have been developed. It was found that toxicity was reduced in doses that had increased levels of monoester alkaloids, while toxicity was directly correlated to an increase in DAs [20, 21].

In our previous work, we reasoned that* P. ginseng* could have a direct effect on the metabolism of these DAs (aconitine, mesaconitine, and hypaconitine) in vitro. Furthermore, we speculated that metabolism stemmed from oxidative transformations mediated by CYP3A4, which was confirmed to be the primary sub-cytochrome P450s (CYPs) for phase 1 metabolism of DAs. And researchers also found that biological activities and pharmacokinetics difference of three representative alkaloids: aconitine, benzoylaconine, and aconine were observed after oral administration in rats [22]. Though aconitine, mesaconitine, and hypaconitine were all belonging to DAs, the structural involvement of sub-CYPs in the metabolism and toxicity of them [23] was not entirely consistent. Zhang Q et al. investigated the pharmacokinetic difference of alkaloids in rats after oral administration of decoction of Aconiti Lateralis Radix. They found that different preparative methods resulted in significant difference on exposure and pharmacokinetic characteristics of alkaloids [24]. Therefore, we wanted to further evaluate whether* P. ginseng* directly influences the metabolism of DAs in vivo, which to the best of our knowledge has yet to be studied.

The present study therefore aims to investigate the pharmacokinetic difference between aconitine, mesaconitine, and hypaconitine after exposure to* P. ginseng* in rats.

## 2. Materials and Methods

### 2.1. Materials and Chemicals

*P. ginseng* was purchased from qiancao Group Co., Ltd. (Beijing, China). Aconiti Lateralis Radix was produced by Tongrentang (Beijing, China). Carbamazepines were purchased from Sigma-Aldrich Co., Ltd. (Darmstadt, Germany). Ginsenoside Rf (CAS:52286-58-5), Notoginsenoside R2 (CAS:80418-25-3), Ginsenoside Rg2 (CAS:52286-74-5), Ginsenoside Rh1 (CAS:63223-86-9), Ginsenoside Rg3 (CAS:14197-60-5), Ginsenoside Rb3 (CAS: 68406-26-8 ), Chikusetsusaponin IVa (51415-02-2), Ginsenoside F2 (CAS:62025-49-4), Ginsenoside Rd (CAS:52705-93-8), Ginsenoside Rg6 (CAS:147419-93-0), Aconitine (CAS: 302-27-2), Mesaconitine (CAS: 2752-64-9), and Hypaconitine (CAS: 6900-87-4) were purchased from EFEBIO Co., Ltd. (Shanghai, China). Acetonitrile and Methanol (HPLC grade) were purchased from Fisher Scientific Company Inc. (Boston, USA).

### 2.2. The Preparation of* P. ginseng* and Aconiti Lateralis Radix Extract

*P. ginseng* (300 g) was soaked in 2.4 L doubly distilled water and boiled for 1 hour. The filtrate was collected and previous step was repeated. The combined filtrates were concentrated by rotary evaporation to afford extracts of 0.5 g/ml (according to the concentration of the crude drugs). The same protocol was used to obtain the extract of Aconiti Lateralis Radix. Extracts were centrifuged at 12,000g for 10 min, the supernatant transferred to a clean centrifuge tube and stored at -20°C.

### 2.3. The Preparation of Reference Solution

Stock solutions were prepared by dissolving accurately weighed reference compounds in methanol at concentrations 1mg/ml individually. Then working solutions were diluted with 50% methanol respectively at concentration of 10 *μ*g/ml, and centrifuged at 12,000 g for 10 min. Then samples were filtered through a 0.22 *μ*m micropore membrane. A 5 *μ*L volume of supernatant was injected into LC-MS system for analysis.

### 2.4. The Qualitative Analysis of Herb Extracts

UPLC/Q-TOF-MS (Waters, USA) was used to detect the target components of herb extracts. The extracts were centrifuged at 12,000g for 10 min, diluted to a specific concentration, and filtered through a 0.22 *μ*m Micropore membrane. A 5 *μ*l injection was used for subsequent analysis.

Chromatographic separation was conducted on Waters ACQUITY UPLC HSS T3 1.8 *μ*m column (2.1 mm × 100 mm) and the column temperature was 45°C. The flow rate was 0.4ml/min. Room temperature was 20 ± 2°C. Phase A consisted of water and 0.1% formic acid (v/v) and phase B consisted of acetonitrile (ACN) 0.1% formic acid (v/v). Column separation for Aconiti Lateralis Radix extract was performed by gradient elution program: 0-1 min, 10% B; 2-25 min, 90% B; 26-28 min, 10% B; 30 min, 10% B. Column separation for* P. ginseng* extract was performed by gradient elution program: 0-1 min, 2.0-20% B; 1-27 min, 20-50% B; 29-30 min, 2% B.

Electrospray ionization (ESI) and positive ion V mode were used to determine the components of Aconiti Lateralis Radix extract. Negative ion V mode was used to determine the components of* P. ginseng* extract. The main parameters for mass spectrum were set as follows: capillary voltage: 3.0 kv; sample cone: 40 v; source temperature: 100°C; desolvation temperature: 350°C; cone gas flow: 450 L/h; desolvation gas flow: 900 L/h; injection volume: 5 *μ*l. The composition identification was done by combining reference substance and molecular weight deviation.

### 2.5. Animals and Treatments

24 male Sprague-Dawley (SD) rats (180-220 g) were obtained from the animal experiment center of the Academy of Military Medical Science of People's license (Beijing, China): SCXK (army)-2012-0004. The environment of raising animals was controlled (room temperature: 22 ± 2°C, humidity: 50 ± 5%, 12 h dark-light cycle). All of the animal experiments were performed in accordance with the guidelines of European community, approved by Institution of Animal Care and Use Committee, Academy of Military Medical Science (IACUC-AMMS). All animals were kept in these environments for 5 days before experiments.

Rats were randomly divided into 4 groups: control group,* P. ginseng* (low, middle, and high) group. 7 days of oral administration of* P. ginseng* extract was administered at a dosage of 0.1, 1, 10 g/kg for* P. ginseng* group (low, middle, and high), respectively, once daily. Distilled water was administered at a dosage of 2 ml/kg for control group, simultaneously. On the 8th day, Aconiti Lateralis Radix extracts (1 g/kg) were orally administered to all groups. 0.5 ml plasma samples were collected in 1.5 ml centrifuge tubes (containing heparin sodium) at 10, 30, 60, 90, 120, 240, 480, 720, 1440, and 2880 min. All plasma samples were centrifuged for 15 min at 4000g. The supernatant was separated to another 1.5 ml centrifuge tubes and stored at -20°C.

### 2.6. Preparation of Plasma Samples and Method Validation

Liquid-liquid extraction (ethyl acetate) and protein precipitation (50% acetonitrile plus 50% methanol) methods were compared to determine the best method to prepare the plasma sample. Liquid-liquid extraction method was chosen, due to reproducibly high recovery rates. The details of the extraction are as follows: 10 *μ*L carbamazepine (IS) was added to 90 *μ*L plasma, spiked with 400 *μ*L ethyl acetate and the mixture vortexed for 15 seconds. After centrifugation at 12,000g for 10 min, the supernatant was transferred to another 1.5 mL centrifuge tube and dried under a stream of nitrogen at 37°C. The residue was redissolved in 100 *μ*L 50% acetonitrile and centrifuged at 12,000g for 10 min and filtered through a 0.22 *μ*m Micropore membrane. A 5 *μ*L volume of supernatant was injected into LC-MS system for analysis.

Plasma from rats without any processing was used to evaluate the detection methods. 0.1, 0.25, 0.5, 1, 2.5, 5, 10 ng/ml were chosen to calculate standards curve. 0.1, 0.4, 4, 8 ng/mL were selected as the QC sample (n = 4) with repeated operations used to calculate accuracy and precision of the detection method. The data was acquired after keeping the samples at either +4°C 24 hours or room temperature (about 25°C) for 24 hours as well as using freeze-thaw methods (three times) to evaluate stability. Recovery of the developed methods was calculated by the following formula: recovery = measured value/ theoretical value × 100%.

### 2.7. Xevo TQ-S Conditions

Xevo TQ-S (Waters, USA) was utilized for the analysis of all biological samples. Chromatographic separation was conducted on Waters ACQUITY UPLC HSS T3 1.8 *μ*m column (2.1 mm × 100 mm). Column temperature was 45°C and the flow rate was 0.5ml/min. Room temperature was 20 ± 2°C. Phase A consisted of water and 0.1% formic acid (v/v), and phase B consisted of acetonitrile (ACN) 0.1% formic acid (v/v). Column separation was performed by gradient elution: 0-0.5 min, 10% B; 1-2 min, 90% B; 4 min, 10% B.

Electrospray ionization (ESI) and positive ion V mode were used to determine the samples. The main parameters for mass spectrum were set as follows: capillary voltage: 3.5 kv; sample cone: 60 v; source offset: 50 v; source temperature: 150°C; desolvation temperature: 350°C; cone gas flow: 150 L/h; desolvation gas flow: 650 L/h; injection volume: 5 *μ*L.

### 2.8. Statistical Analysis

The data was acquired and analyzed by XEVO-QT-S (Waters). The pharmacokinetic parameters were analyzed by DAS pharmacokinetic program (Version 2.0). SAS statistical software was used to determine the difference between each group. All data was presented as mean ± SD, and P<0.05 was considered statistically significant. The figures were processed and displayed by GraphPad prism software (Version 5.01).

## 3. Results

### 3.1. The Qualitative Analysis of Herb Extracts

**Figure 1:**
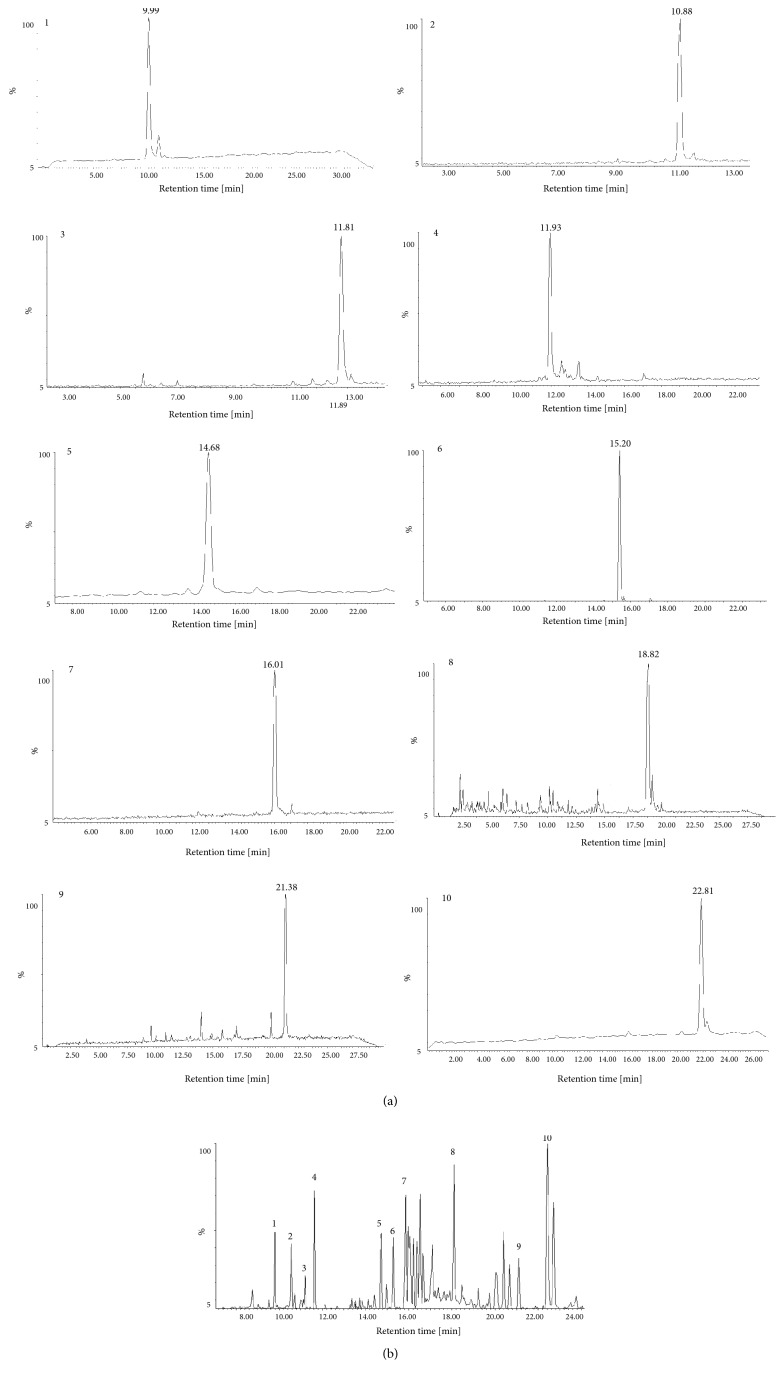
The negative ion peak chromatograms of* P. ginseng* extracts and reference substances of main components by UPLC-Q/TOF-MS. (a) Reference substances and (b)* P. ginseng* extracts (1: ginsenoside Rf; 2: notoginsenoside R2; 3: ginsenoside Rg2; 4: ginsenoside Rh1; 5: ginsenoside Rb3; 6: chikusetsusaponin IVa; 7: ginsenoside Rd; 8: ginsenoside Rg6; 9: ginsenoside F2; 10: ginsenoside Rg3).

**Figure 2:**
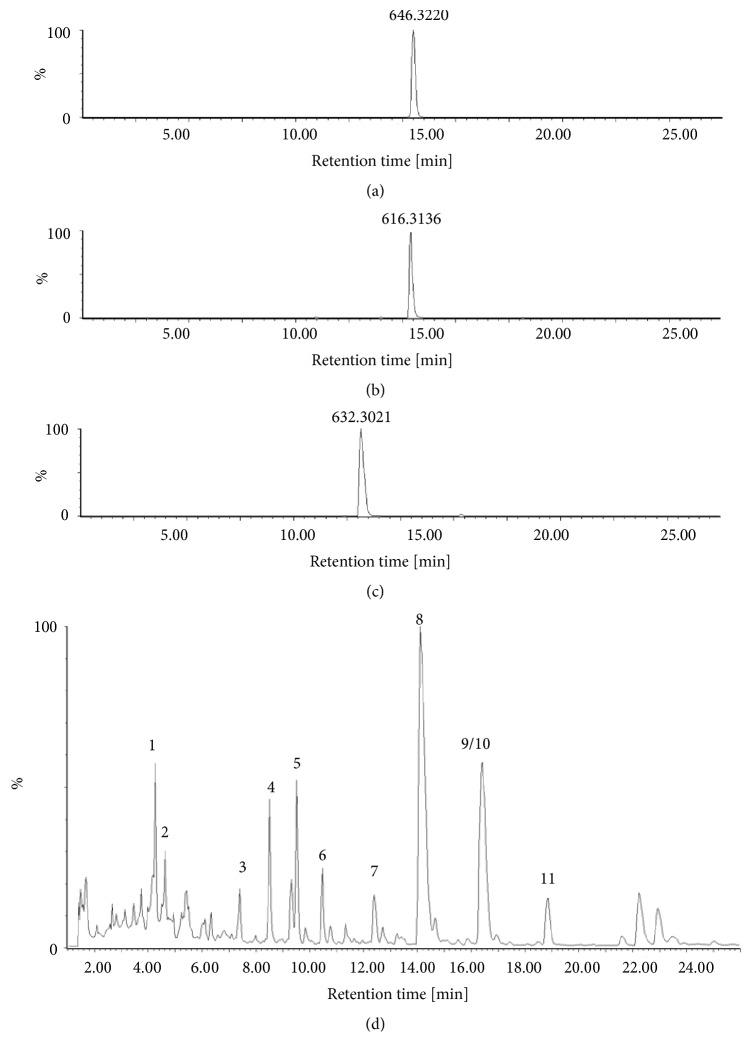
The positive ion peak chromatograms of aconitine, mesaconitine, hypaconitine, and Aconiti Lateralis Radix extracts by UPLC-Q/TOF-MS. (a) aconitine: 646.3220 (m/z); (b) mesaconitine: 632.3021(m/z); (c) hypaconitine: 616.3136 (m/z); and (d) Aconiti Lateralis Radix extracts (1: inositol; 2: fuziline; 3: fuziline; 4: bullatine; 5: talatisamine; 6: benzoylmesaconine; 7: chasmanine; 8: mesaconitine; 9/10: hypaconitine/aconitine; 11: aldohypaconitine).

The main components in* P. ginseng* included ginsenoside Rf, Rg2, Rg3, Rb3, Rh1, Rd, Rg6, F2, notoginsenoside R2, and chikusetsusaponin IVa (Figure 1 and Table 1). The positive ion peak chromatograms of aconitine, mesaconitine, and hypaconitine in Aconiti Lateralis Radix by UPLC-Q/TOF-MS were shown in Figure 2 and clearly show that aconitine, mesaconitine, and hypaconitine were successfully extracted from Aconiti Lateralis Radix. The assigned identity, retention time, molecular formula, theoretical mass/Da, mean measured mass/Da, and mass accuracy (1.0 × 10^−6^) of herb extract were shown in Tables 1 and 2.

### 3.2. Method Validation

#### 3.2.1. Selectivity and Specificity

**Figure 3:**
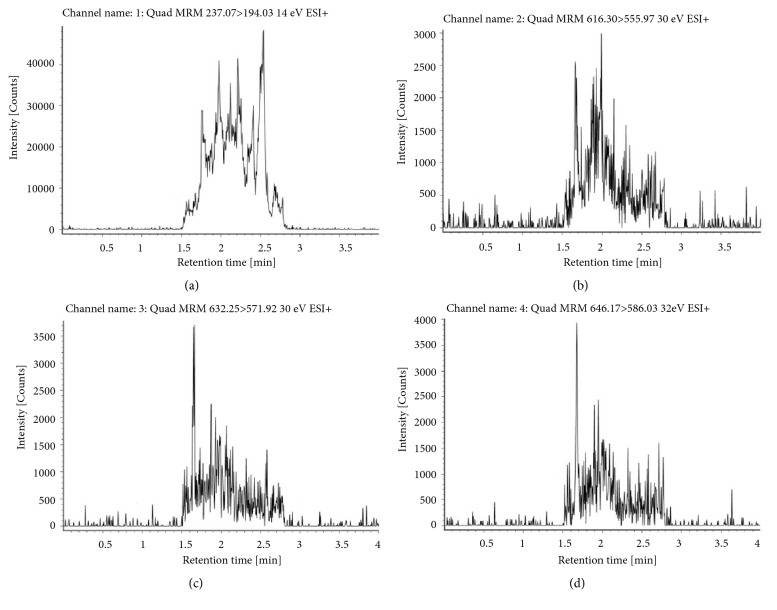
The positive ion peak chromatograms of aconitine, mesaconitine, hypaconitine, and carbamazepine (IS) in blank group by XEVO-QT-S. (a) Carbamazepine, the ion pair 237.07>194.03. (b) Hypaconitine, the ion pair 616.30>555.97. (c) Mesaconitine, the ion pair 632.25>571.92. (d) Aconitine, the ion pair 646.17>586.03.

**Figure 4:**
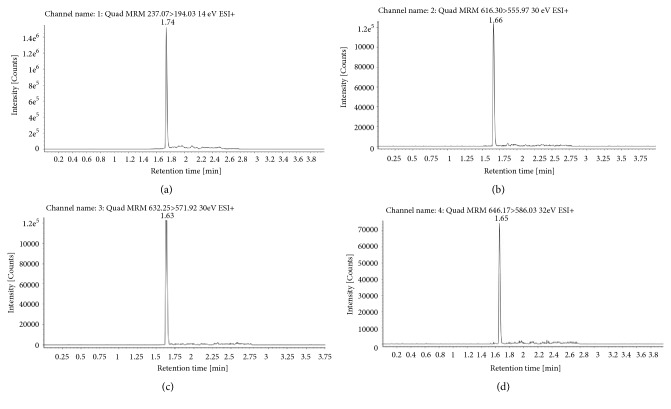
Positive ion peak chromatograms of aconitine, mesaconitine, hypaconitine, and carbamazepine (IS) in biology sample by XEVO-QT-S. (a) Carbamazepine, the ion pair 237.07>194.03. (b) Hypaconitine, the ion pair 616.30>555.97. (c) Mesaconitine, the ion pair 632.25>571.92. (d) Aconitine, the ion pair 646.17>586.03.

The ion pair of relevant compounds were 646.17>586.03 (aconitine), 632.25>571.92 (mesaconitine), and 616.30>555.97 (hypaconitine) with carbamazepine (237.07>194.03) used as the internal standards (IS). The molecular weight and retention time of each compound in the sample are shown in Figure 4, with these peaks not observed for the blank control sample (Figure 3). The limit of detection (LOD) and the lowest limit of quantification (LLOQ) were defined by signal-to-noise ratio method. LOD should be three times of the noise level (S/N >3). LLOQ should be ten times of the noise level (S/N >10). The LOD and LLOQ of three DAs in this experiment were 0.025 ng/ml and 0.1 ng/ml, respectively (Table 3).

#### 3.2.2. Standard Curves and Linearity

**Figure 5:**
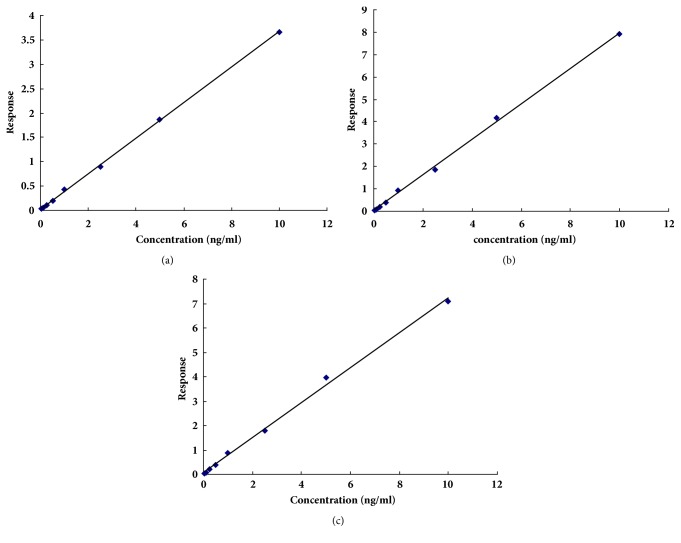
Standard curves of aconitine, mesaconitine, and hypaconitine in biological samples. (a) aconitine, Y = 0.3651X + 0.021, R^2^ = 0.9997; (b) mesaconitine, Y = 0.7952X + 0.0218, R^2^ = 0.9987; (c) hypaconitine, Y = 0.718X + 0.0605, R^2^ = 0.9970.

The standard curve and correlation coefficient of the three components in the selected concentration range 0.1-10 ng/mL are shown in Table 3 and Figure 5.

Three standard curves for aconitine, mesaconitine, and hypaconitine were y = 0.3651x + 0.021, y = 0.7952x + 0.0218, and y = 0.718x + 0.0605, respectively. The correlation coefficient was 0.9997 (aconitine), 0.9987 (mesaconitine), and 0.9970 (hypaconitine).

#### 3.2.3. Precision and Accuracy

The precision and accuracy of the detection of three compounds in both intra- and interday at the linear range are shown in Table 4. Four QC concentrations (0.1, 0.4, 4, 8 ng/mL) were analyzed for each compound. The average precision and accuracy of intraday samples ranged from 2.20% to 7.06% and from 91.18 ± 5.49% to 111.64 ± 2.45%, respectively. The average precision and accuracy of interday ranged from 1.98% to 15.88% and from 83.09 ± 6.75% to 105.48 ± 3.64%, respectively, which proved that the methods had a good accuracy and precision.

#### 3.2.4. Stability and Recovery

The stability data of detected methods were shown in Table 5. The stability of three compounds in 4°C and room temperature (about 25°C) within 24 hours are shown in Table 5(a), with the accuracies ranging from 97.02 ± 9.18% to 101.57 ± 1.29% and from 93.68 ± 2.77% to 100.64 ± 3.60%, respectively. The stability of freeze-thawing the samples is shown in Table 5(b). The RSDs of the accuracy were less than 10.10%. The data displayed that the three compounds had good overall stability.

The recovery of the developed methods was shown in Table 6. The extraction rate for each component at three QC concentrations ranged from 97.74% to 105.02%, and the RSDs ranged from 2.17% to 9.67%.

#### 3.2.5. The Effect of* P. ginseng* on the Metabolism of Aconitine, Mesaconitine, and Hypaconitine in Rats

**Figure 6:**
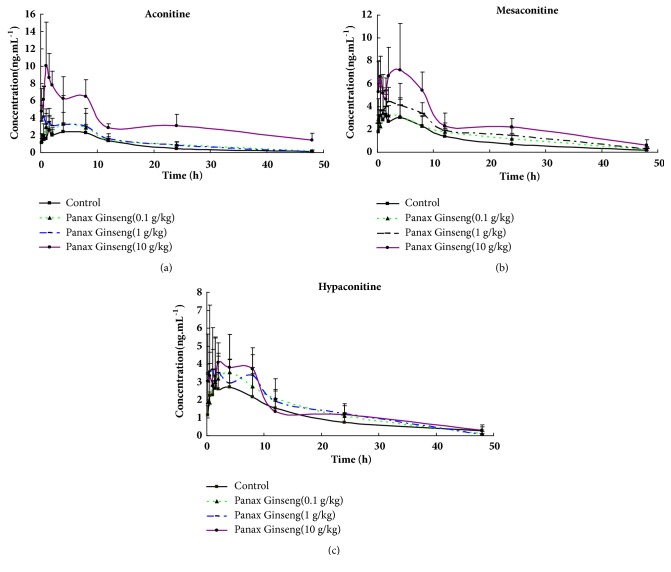
The curves of concentration and time of aconitine, mesaconitine, and hypaconitine in rat's plasma. (a) Aconitine, (b) mesaconitine, and (c) hypaconitine.

The blood concentration-time curves of aconitine, mesaconitine, and hypaconitine are shown in Figure 6. The pharmacokinetic parameters of each compound in the four groups were shown in Tables 7–9. The AUC_(0-t)_ of the three DAs were increased in* P. ginseng* middle group (61.05 ± 12.82 *μ*g/L*∗*h for aconitine, 82.45 ± 18.57 *μ*g/L*∗*h for mesaconitine, and 70.71 ± 7.55 *μ*g/L*∗*h for hypaconitine) and high group (162.98 ± 48.50 *μ*g/L*∗*h for aconitine, 126.07 ± 44.93 *μ*g/L*∗*h for mesaconitine, and 71.75 ± 22.2 *μ*g/L*∗*h for hypaconitine) when compared to the AUC_(0-t)_ of the control group (40.77 ± 9.71 *μ*g/L*∗*h for aconitine, 51.24 ± 21.48 *μ*g/L*∗*h for mesaconitine, and 48.12 ± 24.59 *μ*g/L*∗*h for hypaconitine).

The Vz/F of the DAs in the* P. ginseng* middle group (1458.36 ± 189.64 L/kg for aconitine, 1745.91 ± 488.37 L/kg for mesaconitine, and 1400.22 ± 272.41 L/kg for hypaconitine ) and high group (1547.04 ± 685.53 L/kg for aconitine, 1395.19 ± 472.75 L/kg for mesaconitine, and 2283.28 ± 953.88 L/kg for hypaconitine) were decreased when compared to the control (3345.75 ± 1370.68 L/kg for aconitine, 3146.87 ± 1886.84 L/kg for mesaconitine, and 3881.01 ± 1698.43 for hypaconitine).

The CLz/F of aconitine in all* P. ginseng* groups were low (172.11 ± 35.46 L/h/Kg), middle (169.57 ± 40.27 L/h/Kg), and high (51.09 ± 21.00 L/h/Kg). The CLz/F of mesaconitine and hypaconitine in* P. ginseng *middle (120.75 ± 29.09 L/h/Kg for mesaconitine, 141.34 ± 15.57 L/h/Kg for hypaconitine) and high groups (81.37 ± 29.62 L/h/Kg for mesaconitine, 139.20 ± 37.51 L/h/Kg for hypaconitine) were decreased in comparison to the control group (225.85 ± 54.74 L/h/Kg for aconitine, 217.47 ± 130.54 L/h/Kg for mesaconitine, and 204.16 ± 98.97 L/h/Kg for hypaconitine ).

The C_max_ of aconitine (11.53 ± 3.49 *μ*g/L) and mesaconitine (8.94 ± 2.33 *μ*g/L) increased in* P. ginseng* high groups compared to control (3.88 ± 0.92 *μ*g/L for aconitine, 5.48 ± 2.61 *μ*g/L for mesaconitine), whereas the C_max_ of hypaconitine did not change in all groups. The t_1/2_ of three alkaloids of all groups had no significant difference.

## 4. Discussion

*P. ginseng* and Aconiti Lateralis Radix are widely used in traditional medicine [25] in many Asian countries such as China, Japan, India, and Korea.* P. ginseng*, which displays immune enhancement [26], anticancer [27], anti-inflammatory [28], and antioxidant activities [29], can increase the efficacy of Aconiti Lateralis Radix [3, 25, 30].

Aconiti Lateralis Radix is the root of the Aconite and works to excite central nervous system as well as improve heart function in cases of heart failure [12, 31]. Reports have suggested that Shenfu injection could treat advanced Non-Small-Cell Lung Cancer (NSCLC) [32].

It has also been found that the method of extraction used can produce different concentrations of the active DAs in vitro [33]. Due to the inherent toxicity of DAs, the dosages found in marketed drugs which also contain Aconiti Lateralis Radix are strictly limited according to the 2015 edition of Chinese Pharmacopoeia. It has also been observed that a small change in the concentration of DAs can have a significant influence on the toxicity and efficiency of Aconiti Lateralis Radix [34]. CYP3A4 is the main metabolizing enzymes of CYP450 in human and is responsible for the metabolism of almost 60% endogenous and exogenous substances [35, 36]. In previous research, many ginsenosides, the active component of* P. ginseng*, downregulated CYP3A4 expression through inactivation of human pregnant X receptor (PXR) [4, 37–39]. However, the effect of* P. ginseng* on the metabolism of DAs in vivo has not yet been reported.

Herein, we disclose a refined method to detect DAs in rat blood which contained aconitine, mesaconitine, and hypaconitine. The quantitative concentration range of each component was 0.1-10 ng/mL. The chromatographic behavior of each substance corresponding to signal of MS/MS allowed the simple quantitative and qualitative analysis of each component within the sample. Compared to the previous methods, this method has the advantages of time-saving (the retention time was less than 4.0 min) and low detection concentration, and the sample pretreatment process is simple [11, 24, 40].

In this study, we suggest that* P. ginseng* can inhibit the clearance of aconitine, mesaconitine, and hypaconitine in rats. This result in the accumulation of aconitine, mesaconitine, and hypaconitine within the body may be the basis of DDI between* P. ginseng* and Aconiti Lateralis Radix.

As the key toxic and effective components in Aconiti Lateralis Radix, the concentration of DAs is strictly controlled [41]. Pharmacokinetic changes for DAs when there is combined utilization of* P. ginseng* may lead to a double-sided result. Slowing down the rate of elimination can increase the efficiency of aconitine. However, metabolic inhibition from* P. ginseng* at DAs toxic dose may delay the detoxification, and the concentration range so far is hard to define. Consequently, it is necessary to study the pharmacokinetic changes and concentration range of aconitine, mesaconitine, and hypaconitine for the combined utilization of* P. ginseng* and Aconiti Lateralis Radix. In the next work, we would pay more attention to define the safety of the concentration range of DAs when treating with Aconiti Lateralis Radix. In our previous work, we suggested that* P. ginseng* could downregulate CYP3A4 activities through a combination method. According to the results obtained in this study, we surmise that* P. ginseng* may inhibit the metabolism of small quantities of aconitine, mesaconitine, and hypaconitine in Aconiti Lateralis Radix thus exerting a dramatic change in calcium ion levels which plays a vital role in excitation-contraction coupling in cardiac myocytes.

Researchers have also found that [3] SFI significantly decreased serum CK, LDH, and TNNI3 levels in myocardial ischemia/reperfusion injury (MIRI) rats, while it significantly increased the level of left ventricular systolic pressure (LVSP), left ventricular diastolic pressure (LVDP), maximal rate of the increase of left ventricular pressure (+dp/dtmax), maximal rate of the decrease of left ventricular pressure (-dp/dtmax), left ventricle ejection fraction percentage (EF), and stroke volume (SV).

Aconitine-type alkaloids associated with Aconiti Lateralis Radix efficacy have lower intake and slower elimination in CHF rats, indicating a noninterdependent relationship between its efficacy and toxicity [42]. Coupled with* P. ginseng*, aconitine, mesaconitine, and hypaconitine in Aconiti Lateralis Radix may account for the lower intake and slower elimination in the CHF patient; however, this requires further work to be confirmed.

## 5. Conclusion

A rapid and accurate method to detect the concentration of aconitine, mesaconitine, and hypaconitine in biological samples has been developed and successfully used. From the data obtained in this study, we suggest that* P. ginseng* could inhibit the metabolism of DAs in vivo. Our findings have important implications for* P. ginseng* therapy with medicine containing DAs in clinics.

## Figures and Tables

**Table 1:** Main compounds detected in *P. ginseng* extracts.

Peak no.	Assigned identity	Rt	Molecular formula	[M-H]^−^([M-H+HCOOH]^−^)		
		/min		Theoretical mass/Da	Mean measured mass/Da	Mass accuracy /*∗*10^−6^
1	ginsenoside Rf	9.96	C_42_H_72_O_14_	845.4899	845.495	6.03
2	notoginsenoside R2	10.89	C_41_H_70_O_13_	815.4810	815.4793	-2.08
3	ginsenoside Rg2	11.80	C_42_H_72_O_13_	829.4950	829.4910	-4.82
4	ginsenoside Rh1	11.93	C_36_H_62_O_9_	683.4370	683.4380	1.46
5	ginsenoside Rb3	14.69	C_53_H_90_O_22_	1077.5846	1077.5762	-7.80
6	chikusetsusaponin IVa	15.21	C_42_H_66_O_14_	793.4374	793.4314	-7.56
7	ginsenoside Rd	16.02	C_48_H_82_O_18_	991.3988	991.3899	-8.98
8	ginsenoside Rg6	18.82	C_42_H_70_O_12_	811.4844	811.4838	-0.74
9	ginsenoside F2	21.36	C_42_H_72_O_13_	783.4895	783.4824	-9.06
10	ginsenoside Rg3	22.88	C_42_H_72_O_13_	783.4895	783.4904	1.15

**Table 2:** Main compounds detected in Aconiti Lateralis Radix extracts.

Peak no.	Assigned identity	Rt	Molecular formula	[M+H]^+^		
		/min		Theoretical mass/Da	Mean measured mass/Da	Mass accuracy /×10^−6^
1	inositol	0.51	C_6_H_12_O_6_	180.0643	180.0648	-2.78
2	fuziline	3.01	C_24_H_39_NO_7_	454.2805	454.2811	-1.32
3	fuziline	3.15	C_24_H_39_NO_7_	454.2805	454.2837	-7.04
4	bullatine	3.82	C_24_H_39_NO_6_	438.2856	438.2819	8.44
5	talatisamine	4.05	C_24_H_39_NO_5_	422.2906	422.2905	0.24
6	benzoylmesaconine	8.32	C_31_H_43_NO_10_	590.2695	590.2696	-0.17
7	chasmanine	9.51	C_25_H_41_NO_6_	452.3012	452.3055	-9.51
8	mesaconitine	12.61	C_33_H_45_NO_11_	632.3071	632.3021	7.91
9	hypaconitine	15.92	C_33_H_45_NO_10_	616.3122	616.3136	-0.65
10	aconitine	15.93	C_34_H_47_NO_11_	646.3227	646.3220	1.08
11	aldohypaconitine	18.05	C_33_H_43_NO_11_	630.2914	630.2971	-9.04

**Table 3:** Standard curves, linear range, correlation coefficient, LOD, and LLOQ of aconitine, mesaconitine, and hypaconitine in rat plasma.

Name	Standard curves	Linear range	R^2^	LLOQ	LOD
		(ng/mL)		(ng/mL)	(ng/mL)
Aconitine	y = 0.3651x + 0.0210	0.1-10	0.9997	0.1	0.025
Mesaconitine	y = 0.7952x + 0.0218	0.1-10	0.9987	0.1	0.025
Hypaconitine	y = 0.718x + 0.0605	0.1-10	0.9970	0.1	0.025

**Table 4:** Intra- and Interday precision and accuracy of these standard curves.

Name	QC conc.	Intraday			Interday		
		Concentration	RSD	Accuracy	Concentration	RSD	Accuracy
	(ng/mL)	(ng/mL)	(%)	(%)	(ng/mL)	(%)	(%)
Aconitine	0.10	0.09 ± 0.01	6.03	91.18 ± 5.49	0.08 ± 0.01	8.12	83.09 ± 6.75
	0.40	0.39 ± 0.01	3.31	98.05 ± 3.24	0.40 ± 0.02	4.06	100.49 ± 4.08
	4.00	4.30 ± 0.30	7.06	107.38 ± 7.58	4.00 ± 0.08	1.98	99.92 ± 1.98
	8.00	8.02 ± 0.36	4.45	100.25 ± 4.46	7.94 ± 0.19	2.35	99.28 ± 2.34

Mesaconitine	0.10	0.11 ± 0.00	2.20	111.64 ± 2.45	0.10 ± 0.02	15.88	100.71 ± 15.99
	0.40	0.40 ± 0.01	3.73	100.26 ± 3.74	0.38 ± 0.03	7.86	95.08 ± 7.47
	4.00	3.96 ± 0.23	5.76	99.08 ± 5.7	3.98 ± 0.24	6.10	99.44 ± 6.06
	8.00	7.93 ± 0.28	3.57	99.07 ± 3.54	7.94 ± 0.25	3.15	99.30 ± 3.13

Hypaconitine	0.10	0.10 ± 0.01	5.79	104.52 ± 6.05	0.11 ± 0.00	3.45	105.48 ± 3.64
	0.40	0.38 ± 0.01	3.39	95.55 ± 3.24	0.39 ± 0.01	3.82	97.76 ± 3.74
	4.00	4.17 ± 0.27	6.36	104.26 ± 6.63	4.06 ± 0.18	4.51	101.58 ± 4.58
	8.00	8.02 ± 0.36	4.45	100.25 ± 4.46	7.94 ± 0.19	2.35	99.28 ± 2.34

**Table:** (a) The stability at 4°C and room temperature of detected methods.

Name	QC conc.	4°C temperature stability (24 h)			Room temperature stability (24 h)		
	(ng/mL)	Concentration (ng/mL)	RSD (%)	Accuracy (%)	Concentration (ng/mL)	RSD (%)	Accuracy (%)
Aconitine	0.40	0.40 ± 0.01	2.16	99.65 ± 2.16	0.40 ± 0.01	2.51	100.49 ± 2.52
	4.00	3.96 ± 0.4	10.10	98.98 ± 9.99	3.85 ± 0.13	3.38	96.29 ± 3.26
	8.00	8.13 ± 0.1	1.27	101.57 ± 1.29	7.81 ± 0.36	4.64	97.66 ± 4.54

Mesaconitine	0.40	0.40 ± 0.03	8.71	100.28 ± 8.73	0.39 ± 0.03	7.21	96.34 ± 6.95
	4.00	3.88 ± 0.37	9.47	97.02 ± 9.18	3.75 ± 0.11	2.95	93.68 ± 2.77
	8.00	7.95 ± 0.37	4.59	99.43 ± 4.57	8.06 ± 0.38	4.74	100.72 ± 4.78

Hypaconitine	0.40	0.39 ± 0.01	2.41	97.44 ± 2.35	0.40 ± 0.01	2.51	100.49 ± 2.52
	4.00	4.20 ± 0.22	5.23	104.96 ± 5.49	3.95 ± 0.17	4.35	98.72 ± 4.30
	8.00	8.09 ± 0.39	4.76	101.13 ± 4.82	8.05 ± 0.29	3.58	100.64 ± 3.60

**Table:** (b) The stability at freeze-thaw of detected methods.

Name	QC conc.	Freeze-thaw		
	(ng/mL)	Concentration (ng/mL)	RSD (%)	Accuracy (%)
Aconitine	0.40	0.40 ± 0.01	2.06	99.71 ± 2.06
	4.00	4.00 ± 0.25	6.24	99.91 ± 6.23
	8.00	8.02 ± 0.15	1.87	100.22 ± 1.87

Mesaconitine	0.40	0.38 ± 0.03	7.69	95.09 ± 7.31
	4.00	4.01 ± 0.36	8.89	100.37 ± 8.92
	8.00	7.97 ± 0.27	3.36	99.59 ± 3.35

Hypaconitine	0.40	0.40 ± 0.01	2.06	99.71 ± 2.06
	4.00	4.01 ± 0.24	6.01	100.26 ± 6.03
	8.00	8.19 ± 0.26	3.12	102.33 ± 3.19

**Table 6:** Recovery of the developed methods.

Name	QC conc. (ng/mL)	Recovery (Mean ± SD)	RSD (%)
Aconitine	0.40	99.29 ± 3.88	3.90
	4.00	104.26 ± 9.40	9.02
	8.00	102.00 ± 6.29	6.17

Mesaconitine	0.40	101.67 ± 9.83	9.67
	4.00	105.02 ± 8.19	7.80
	8.00	98.93 ± 5.25	5.30

Hypaconitine	0.40	97.74 ± 3.07	3.14
	4.00	104.56 ± 6.28	6.01
	8.00	103.32 ± 2.24	2.17

**Table 7:** Effect of *P. ginseng* on the pharmacokinetics of aconitine at day 7 after administration.

Aconitine	Control	P. ginseng-low	P. ginseng-middle	P. ginseng-high
AUC_(0-t)_ (*μ*g/L*∗*h)	40.77 ± 9.71	58 ± 13.02	61.05 ± 12.82^*∗*^	162.98 ± 48.50^*∗∗*^
AUC_(0-*∞*)_ (*μ*g/L*∗*h)	46.20 ± 9.57	60.32 ± 12.99	61.49 ± 13.16^*∗*^	228.56 ± 100.03^*∗∗*^
t_1/2z_ (h)	10.71 ± 4.78	10.10 ± 3.72	6.14 ± 1.18	26.49 ± 23.27
T_max_ (h)	2.67 ± 2.91	4.00 ± 3.27	2.11 ± 2.97	1.33 ± 0.41
V_z_/F (L/kg)	3345.75 ± 1370.68	2480.3 ± 944.53	1458.36 ± 189.64^*∗∗*^	1547.04 ± 685.53^*∗∗*^
CL_z_/F (L/h/Kg)	225.85 ± 54.74	172.11 ± 35.46^*∗*^	169.57 ± 40.27^*∗∗*^	51.09 ± 21.00^*∗∗*^
C_max_ (*μ*g/L)	3.88 ± 0.92	5.03 ± 3.02	5.79 ± 2.80	11.53 ± 3.49^*∗∗*^

Note: Compared with the control group, ^*∗*^P<0.05, ^*∗∗*^P<0.01.

**Table 8:** Effect of *P. ginseng* on the pharmacokinetics of mesaconitine at day 7 after administration.

Mesaconitine	Control	P. ginseng-low	P. ginseng-middle	P. ginseng-high
AUC_(0-t)_ (*μ*g/L*∗*h)	51.24 ± 21.48	65.53 ± 9.06	82.45 ± 18.57^*∗*^	126.07 ± 44.93^*∗∗*^
AUC_(0-*∞*)_ (*μ*g/L*∗*h)	58.66 ± 27.59	71.36 ± 12.85	87.1 ± 21.80^*∗*^	138.96 ± 55.36^*∗∗*^
t_1/2z_ (h)	13.17 ± 10.45	12.12 ± 6.11	10.31 ± 3.00	12.56 ± 4.48
T_max_ (h)	1.58 ± 1.32	0.89 ± 0.60	1.83 ± 1.21	2.20 ± 1.98
V_z_/F (L/kg)	3146.87 ± 1886.84	2386.47 ± 832.39	1745.91 ± 488.37^*∗*^	1395.19 ± 472.75^*∗∗*^
CL_z_/F (L/h/Kg)	217.47 ± 130.54	144.13 ± 26.77	120.75 ± 29.09^*∗*^	81.37 ± 29.62^*∗∗*^
C_max_ (*μ*g/L)	5.48 ± 2.61	4.66 ± 1.03	6.02 ± 1.63	8.94 ± 2.33^*∗*^

Note: Compared with the control group, ^*∗*^P<0.05, ^*∗∗*^P<0.01.

**Table 9:** Effect of *P. ginseng* on the pharmacokinetics of hypaconitine at day 7 after administration.

Hypaconitine	Control	P. ginseng-low	P. ginseng-middle	P. ginseng-high
AUC_(0-t)_ (*μ*g/L*∗*h)	48.12 ± 24.59	65.64 ± 13.14	70.71 ± 7.55^*∗*^	71.75 ± 22.2^*∗*^
AUC_(0-*∞*)_ (*μ*g/L*∗*h)	60.16 ± 28.03	70.68 ± 8.89^*∗*^	71.49 ± 8.07^*∗*^	77.26 ± 25.15^*∗*^
t1/2z (h)	14.11 ± 5.41	9.70 ± 3.55	6.97 ± 1.77	11.67 ± 4.21
Tmax (h)	2.67 ± 2.91	3.61 ± 3.47	2.53 ± 3.01	1.83 ± 1.29
Vz/F (L/kg)	3881.01 ± 1698.43	2023.85 ± 818.73	1400.22 ± 272.41^*∗*^	2283.28 ± 953.88^*∗*^
CLz/F (L/h/Kg)	204.16 ± 98.97	143.1 ± 15.52	141.34 ± 15.57^*∗*^	139.20 ± 37.51^*∗*^
Cmax (*μ*g/L)	4.63 ± 1.96	4.02 ± 2.01	5.85 ± 2.71	4.87 ± 1.36

Note: Compared with the control group, ^*∗*^P<0.05, ^*∗∗*^P<0.01.

## Data Availability

The data used to support the findings of this study are available from the corresponding author upon request.

## Abbreviations

DAs: Diester Alkaloids
*P. ginseng*: * Panax ginseng*
UPLC-Q/TOF-MS: Ultra Performance Liquid Chromatography time-off-flight mass spectrometer
ESI: Electro spray ionization
DDI: Drug-drug interaction
SD: Sprague-Dawley
ACN: Acetonitrile
MIRI: Myocardial ischemia/reperfusion injury
NGF: Nerve growth factor
LVSP: Left ventricular systolic pressure
LVDP: Left ventricular diastolic pressure
+dp/dtmax: Maximal rate of the increase of left ventricular pressure
-dp/dtmax: Maximal rate of the decrease of left ventricular pressure
EF: Left ventricle ejection fraction percentage
SFI: Shenfu injection
LOD: The limit of detection
LLOQ: The lowest limit of quantification.
